# Control of hyperglycaemia in paediatric intensive care (CHiP): study protocol

**DOI:** 10.1186/1471-2431-10-5

**Published:** 2010-02-05

**Authors:** Duncan Macrae, John Pappachan, Richard Grieve, Roger Parslow, Simon Nadel, Margrid Schindler, Paul Baines, Peter-Marc Fortune, Zdenek Slavik, Allan Goldman, Ann Truesdale, Helen Betts, Elizabeth Allen, Claire Snowdon, Deborah Percy, Michael Broadhead, Tara Quick, Mark Peters, Kevin Morris, Robert Tasker, Diana Elbourne

**Affiliations:** 1 Paediatric Intensive Care Unit, Royal Brompton Hospital, Sydney Street, London SW3 6NP, UK; 2 Paediatric Intensive Care Unit, Southampton University Hospitals NHS Trust, Tremona Road, Southampton SO16 6YD, UK; 3 Health Services Research Unit, London School of Hygiene and Tropical Medicine, Keppel Street, London WC1E 7HT, UK; 4 Paediatric Epidemiology Group Centre for Epidemiology & Biostatistics, 8,49 Worsley Building University of Leeds, Leeds LS2 9JT, UK; 5 Paediatric Intensive Care Unit, Imperial Healthcare NHS Trust, St Mary's Hospital, London, WC2, UK; 6 Paediatric Intensive Care Unit, Bristol Royal Hospital for Children, Bristol BS2 8BJ, UK; 7 Paediatric Intensive Care Unit, Alder Hey Children's Hospital, Eaton Rd, Liverpool L12 2AP, UK; 8 Paediatric Intensive Care Unit, Royal Manchester Children's Hospital, Hospital Road, Pendlebury, Manchester M27 4HA, UK; 9 Royal Brompton Hospital, Sydney Street, London, SW3 6NP, UK; 10 Cardiac Intensive Care Unit, Great Ormond Street Children's Hospital, London, WC1 3JH, UK; 11 Medical Statistics Unit, London School of Hygiene and Tropical Medicine, Keppel Street, London WC1E 7HT, UK; 12 Paediatric Intensive Care Unit, Royal Brompton Hospital, Sydney Street, London, SW3 6NP, UK; 13 Medical Statistics Unit, London School of Hygiene and Tropical Medicine, Keppel Street, London WC1E 7HT, UK; 14 London School of Hygiene and Tropical Medicine, Keppel Street, London WC1E 7HT, UK; 15 Medical Statistics Unit, London School of Hygiene and Tropical Medicine, Keppel Street, London WC1E 7HT, UK; 16 Great Ormond Street Children's Hospital, London, WC1N 3JH, UK; 17 Parent, c/o London School of Hygiene and Tropical Medicine, Keppel Street, London WC1E 7HT, UK; 18 Paediatric Intensive Care Unit, Great Ormond Street Children's Hospital, London, WC1 3JH, UK; 19 Paediatric Intensive Care Unit, Birmingham Children's Hospital NHS Foundation Trust, Steelhouse Lane, Birmingham B46NH, UK; 20 Department of Paediatrics, University of Cambridge, Cambridge, B2 0QQ, UK; 21 Medical Statistics Unit, London School of Hygiene and Tropical Medicine, Keppel Street, London WC1E 7HT, UK

## Abstract

**Background:**

There is increasing evidence that tight blood glucose (BG) control improves outcomes in critically ill adults. Children show similar hyperglycaemic responses to surgery or critical illness. However it is not known whether tight control will benefit children given maturational differences and different disease spectrum.

**Methods/Design:**

The study is an randomised open trial with two parallel groups to assess whether, for children undergoing intensive care in the UK aged ≤ 16 years who are ventilated, have an arterial line in-situ and are receiving vasoactive support following injury, major surgery or in association with critical illness in whom it is anticipated such treatment will be required to continue for at least 12 hours, tight control will increase the numbers of days alive and free of mechanical ventilation at 30 days, and lead to improvement in a range of complications associated with intensive care treatment and be cost effective.

Children in the tight control group will receive insulin by intravenous infusion titrated to maintain BG between 4 and 7.0 mmol/l. Children in the control group will be treated according to a standard current approach to BG management.

Children will be followed up to determine vital status and healthcare resources usage between discharge and 12 months post-randomisation. Information regarding overall health status, global neurological outcome, attention and behavioural status will be sought from a subgroup with traumatic brain injury (TBI).

A difference of 2 days in the number of ventilator-free days within the first 30 days post-randomisation is considered clinically important. Conservatively assuming a standard deviation of a week across both trial arms, a type I error of 1% (2-sided test), and allowing for non-compliance, a total sample size of 1000 patients would have 90% power to detect this difference. To detect effect differences between cardiac and non-cardiac patients, a target sample size of 1500 is required. An economic evaluation will assess whether the costs of achieving tight BG control are justified by subsequent reductions in hospitalisation costs.

**Discussion:**

The relevance of tight glycaemic control in this population needs to be assessed formally before being accepted into standard practice.

**Trial Registration:**

Current Controlled Trials ISRCTN61735247

## Background

The ability to control blood sugar is known to be impaired in patients subjected to the stress of major surgery or critical illness resulting in high blood sugar levels (hyperglycaemia)[1]. This may in part result from insulin resistance, as insulin-dependent glucose uptake has been shown to be reduced in various organs and tissues during critical illness. Glucose uptake is however increased in non-insulin dependent tissues such as brain, red blood cells and wounds. This imbalance of glucose metabolism has previously been interpreted as the body's plea for tolerating moderately high levels of glucose during critical illness and injury and treatment of 'stress-induced' hyperglycaemia has typically only been initiated if BG levels are persistently and substantially elevated.

### Hyperglycaemia in Critically Ill Adults

Over recent years several studies have associated hyperglycaemia with adverse outcomes during acute illness in adults:

#### Myocardial infarction

In a meta-analysis [2], patients with acute myocardial infarction without diabetes who had glucose concentrations more than or equal to range 6.1-8.0 mmol/L had a 3.9-fold (95% CI 2.9-5.4) higher risk of death than patients without diabetes who had lower glucose concentrations. Glucose concentrations higher than values in the range of 8.0-10.0 mmol/L on admission were associated with increased risk of congestive heart failure or cardiogenic shock in patients without diabetes. Stress hyperglycaemia with myocardial infarction is associated with an increased risk of in-hospital mortality and increased risk of congestive heart failure or cardiogenic shock in patients without diabetes.

#### Stroke

Capes et al. conducted a systematic review and meta-analysis of the literature relating acute post stroke glucose levels to the subsequent course [3]. A comprehensive literature search was done for cohort studies reporting mortality and/or functional recovery after stroke in relation to admission glucose level. Thirty-two studies were identified for which pre-defined outcomes could be analysed in 26. After stroke, the unadjusted relative risk of in-hospital or 30-day mortality associated with admission glucose level >6 to 8 mmol/L was 3.07 (95% CI, 2.50 to 3.79) in non-diabetic patients and 1.30 (95% CI, 0.49 to 3.43) in diabetic patients. Non-diabetic stroke survivors whose admission glucose level was >6.7 to 8 mmol/L also had a greater risk of poor functional recovery (relative risk = 1.41; 95% CI, 1.16 to 1.73).

#### Head injury and multi-system trauma

Hyperglycaemia has been shown to be an independent predictor of poor outcome in adult patients[4] and children with head injury[5,6] and multiple trauma[7].

#### Pulmonary function

Hyperglycaemia has been shown to be associated with diminished pulmonary function in adults even in the absence of diabetes mellitus[8] and a range of other effects with potential to injure the lung[9].

#### Gastrointestinal effects

Hyperglycaemia has been shown to be associated with delayed gastric emptying[10], decreased small bowel motility and to increase sensation and cerebral evoked potentials to a range of gastrointestinal stimuli in adult volunteers [11-14].

#### Infections

*In vitro *responsiveness of leukocytes stimulated by inflammatory mediators is inversely correlated with glycaemic control[15]. This reduction in polymorphonuclear leucocyte responsiveness may contribute to the compromised host defence associated with sustained hyperglycaemia[15], and indeed, hyperglycaemia has been shown to be associated with an increased rate of serious infections after adult cardiac[16] and vascular[17] surgery.

### Studies of Control of Glycaemia in Adults

Recent reports from adult populations suggest that control of glycaemia during acute illness can be associated with improved outcomes[18-22].

Furnary[21] studied the hypothesis that since hyperglycaemia was associated with higher sternal wound infection rates following adult cardiac surgery, aggressive control of glycaemia might lead to lower infection rates. In a prospective study of 2,467 consecutive diabetic patients who underwent open heart surgical procedures, patients were classified into two sequential groups. A control group included 968 patients treated with sliding-scale-guided intermittent subcutaneous insulin injections. A study group included 1,499 patients treated with a continuous intravenous insulin infusion in an attempt to maintain a BG level of less than 11.1 mmol/l. Compared with subcutaneous insulin injections, continuous intravenous insulin infusion induced a significant reduction in perioperative BG levels, which led to a significant reduction in the incidence of deep sternal wound infection in the continuous intravenous insulin infusion group (0.8% [12 of 1,499]) versus the intermittent subcutaneous insulin injection group (2.0% [19 of 968], p = 0.01). The use of perioperative continuous intravenous insulin infusion in diabetic patients undergoing open heart surgical procedures appears to significantly reduce the incidence of major infections.

Malmberg[19] randomly allocated patients with diabetes mellitus and acute myocardial infarction to intensive insulin therapy (n = 306) or standard treatment (controls, n = 314). The mean (range) follow up was 3.4 (1.6-5.6) years. There were 102 (33%) deaths in the treatment group compared with 138 (44%) deaths in the control group (relative risk (95% confidence interval) 0.72 (0.55 to 0.92); p = 0.011). The effect was most pronounced among the predefined group that included 272 patients without previous insulin treatment and at a low cardiovascular risk (0.49 (0.30 to 0.80); p = 0.004). Intensive insulin therapy improved survival in diabetic patients with acute myocardial infarction. The effect seen at one year continued for at least 3.5 years, with an absolute reduction in mortality of 11%.

In 2001 Van den Berghe and colleagues from Leuven, Belgium[18] reported the results of a randomised trial in adults undergoing intensive care following surgical procedures. This trial showed that the use of insulin to tightly control BG led to a reduction in mortality (32%), mean length of intensive care stay (22%), and significantly lower occurrence of a range of complications of critical illness such as renal failure, infection, inflammation, anaemia and polyneuropathy. Duration of intensive care stay was 3.4 days shorter in the insulin group.

Recently the Leuven group[22] have reported that, in addition to adult surgical intensive care patients, intensive insulin therapy reduces morbidity in adults who require intensive care for treatment of medical conditions. In this prospective randomised controlled trial, patients were randomly assigned to a regime of strict normalisation of BG (4.4-6.1 mmol/l) with use of insulin, or conventional therapy where insulin is administered only when BG levels exceeded 12 mmol/l, with the infusion tapered when the level fell below 10 mmol/l. In the intention to treat analysis of the 1200 patients included, ICU and in-hospital mortality were not significantly altered by intensive insulin therapy, however for those patients requiring more than 3 days intensive care, mortality was significantly reduced from 52.5 to 43% (p = 0.009). Morbidity was significantly reduced by intensive insulin therapy with a lower incidence of renal injury and shorter length of mechanical ventilation and duration of hospital stay noted. Beyond the fifth day of intensive insulin therapy, all morbidity endpoints were beneficially affected, whereas for those patients staying less than 3 days, none of the morbidity end-points were significantly different between the two treatment groups.

On the basis of these studies, several groups have recommended that tight glycaemic control with intensive insulin therapy become a standard of care for the critically ill adult patients. The Joint Commission on Accreditation of Healthcare Organization (JACHO) recently proposed tight glucose control for the critically ill as a core quality of care measure for all U.S. hospitals that participate in the Medicare program[23]. The Institute for Healthcare Improvement, together with an international initiative by several professional societies including the American Thoracic Society, is promoting a care "bundle" for severe sepsis that also includes intensive glycaemic control for critically ill adults[24]. Both the Society of Critical Care Medicine and European Society of Intensive Care Medicine have incorporated TGC into their recently publicised 'Surviving Sepsis' guidelines. These initiatives represent important attempts to translate research findings into improved care at the bedside[25].

The possible mechanisms by which different glucose control strategies might influence clinical outcomes are yet to be fully elucidated. There is a substantial body of published research which points to an association between hyperglycaemia and organ/tissue dysfunction. In models of both focal and global cerebral ischaemia, hyperglycaemia has been shown to be associated with exacerbation of intracellular acidosis[26-28], accumulation of extracellular glutamate[29], cerebral oedema formation and disruption[30]of the blood-brain barrier[31]. In ischaemic brain injury, hyperglycaemia may worsen injury by promoting anaerobic metabolism and consequent intracellular acidosis. In the rat myocardium, hyperglycaemia leads to up-regulation of inducible nitric oxide synthase, resulting ultimately in an increase in production of superoxide, a condition favouring the production of the powerful pro-oxidant peroxynitrite. This highly reactive free radical has the power to cause direct oxidant damage to myocardial cells or to induce myocardial cell apoptosis[32,33]. Similar adverse mechanisms have been shown to exist in hyperglycaemic patients [34,35]. Improved clinical outcomes may arise not necessarily solely as a result of control of BG. Insulin lowers free fatty acids and normalises endothelial function[36], is associated with anabolic effects[37,38], has been shown to have anti-inflammatory effects[39,40] and to have cardio-protective effects[41], all of which may contribute independently to better outcomes in critical illness.

### Hyperglycaemia in Critically Ill Children

Over 10,000 children are admitted to intensive care units in England and Wales each year[42]. Hyperglycaemia, defined as BG > 7 mmol/l, occurs frequently during critical illness or after major surgery in children, with a reported incidence of up to 86%[43]. As in adults, the occurrence of hyperglycaemia has been shown to be associated with poorer outcomes including death, sepsis, and longer length of intensive care stay in critically ill children[43-46]. Non-randomised research in children includes a number of reports from general[43-45,47] and cardiac PICUs[46] showing that high BG levels occur frequently in critically ill children and that BG levels are significantly higher in children who die than in children who survive.

Srinivasan[43] studied the association of timing, duration, and intensity of hyperglycaemia with PICU mortality in critically ill children. The study was a retrospective, cohort design and included 152 critically ill children receiving vasoactive infusions or mechanical ventilation. Peak BG of > 7 mmol/L occurred in 86% of patients. Compared with survivors, non-survivors had higher peak BG (17.3 mmol/L +/- 6.4 vs. 11.4 +/- 4.4 mmol/L, p <.001). Non-survivors had more intense hyperglycaemia during the first 48 hrs in the PICU (7 +/-2.1 mmol/L) vs. survivors (6.4 +/- 1.9 mmol/L, p <.05). Univariate logistic regression analysis showed that peak BG and the duration and intensity of hyperglycaemia were each associated with PICU mortality (p <.05). Multivariate modelling controlling for age and Paediatric Risk of Mortality scores showed independent association of peak BG and duration of hyperglycaemia with PICU mortality (p <.05). This study demonstrated that hyperglycaemia is common among critically ill children. Peak BG and duration of hyperglycaemia appear to be independently associated with mortality. The study was limited by its retrospective design, its single-centre location and the absence of cardiac surgical cases, a group which make up approximately 40% of paediatric intensive care (PICU) admissions in the UK.

Halverson-Steele[46] has recently shown in a retrospective study, that hyperglycaemia was associated with poor outcomes in 526 children following cardiac surgery. Nineteen patients (3.6%) died postoperatively (median 11 days, range 1-17 days). Peak plasma glucose concentrations in survivors (mean 10.7 mmol/l, SD 3.7) was significantly lower than the peak value recorded in non-survivors (mean 14.3 mmol/l, SD 4.2; p = 0.0017). The 147 patients who were discharged from ICU within 24 hours had lower plasma glucose concentrations on admission (mean 7.5 mmol/l, SD 2.3) and peak plasma glucose concentrations (mean 9.2 mmol/l, SD 2.3) than the remaining patients staying longer than 24 hours (mean 8.1 mmol/l, SD 4.0; p = 003 and mean 11.3 mmol/l, SD 3.9; p < 0.0001, respectively). Peak plasma glucose concentrations were also lower in 387 patients admitted for up to 5 days (mean 10.1 mmol/l, SD 2.9) when compared with those patients with ICU stays of > 5 days (mean 12.7 mmol/l/, SD 4.6; p < 0.0001).

Hall[45] investigated the incidence of hyperglycaemia in infants with necrotizing enterocolitis (NEC) and the relationship between glucose levels and outcome in these infants. Glucose measurements (n = 6508) in 95 neonates with confirmed NEC admitted to the surgical intensive care unit were reviewed. Glucose levels ranged from 0.5 to 35.0 mmol/L. 69% of infants became hyperglycaemic (>8 mmol/L) during their admission. Thirty-two infants died. Mortality rate tended to be higher in infants when maximal glucose concentration exceeded 11.9 mmol/L compared with those with maximum glucose concentrations of less than 11.9 mmol/L, and late (>10 days admission) mortality rate was significantly higher in these infants (29% v, 2%; p = .0009). Linear regression analysis indicated that maximum glucose concentration was significantly related to length of stay (p <.0001).

Branco[44] showed that there is an association between hyperglycaemia and increased mortality in children with septic shock. They prospectively studied all children admitted to a regional PICU with septic shock refractory to fluid therapy over a period of 32 months. The peak glucose level in those with septic shock was 11.9 +/- 5.4 mmol/L (mean +/- SD), and the mortality rate was 49.1% (28/57). In non-survivors, the peak glucose level was 14.5 +/- 6.1 mmol/L, which was higher (p <.01) than that found in survivors (9.3 +/- 3.0 mmol/L). The relative risk of death in patients with peak glucose levels of ≥ 9.9 mmol/L was 2.59 (range, 1.37-4.88).

Faustino[47] demonstrated that hyperglycaemia occurs frequently among critically ill non-diabetic children and is associated with higher mortality and longer lengths of stay. They performed a retrospective cohort study of 942 non-diabetic patients admitted to a PICU over a 3 year period. The prevalence of hyperglycaemia was based on initial PICU glucose measurement, highest value within 24 hours, and highest value measured during PICU stay up to 10 days after the first measurement. Through the use of three cut-off values (6.7 mmol/L, 8.3 mmol/L, and 11.1 mmol/L), the prevalence of hyperglycaemia was 16.7% to 75.0%. The relative risk (RR) for dying increased for maximum glucose within 24 hours >8.3 mmol/L (RR, 2.50; 95% confidence interval (CI), 1.26 to 4.93) and highest glucose within 10 days >6.7 mmol/L (RR, 5.68; 95% CI, 1.38 to 23.47).

Pham[48] have recently reported their experience of adopting a policy of 'intensive' insulin therapy to achieve BG levels 5 mmol/L to 6.7 mmol/L. They reviewed the records of children with ≥ 30% total body surface area burn injury admitted over a 3 year period. The first cohort of 31 children received 'conventional insulin therapy', whilst the subsequent cohort of 33 children received 'intensive insulin therapy'. The demographic characteristics and injury severity were similar between the groups. Intensive insulin therapy was positively associated with survival and a reduced incidence of infections. The authors therefore concluded that intensive insulin therapy to maintain normoglycaemia in severely burned children could be safely and effectively implemented in a paediatric burns unit and that this therapy seemed to lower infection rates and improve survival.

There is therefore mounting evidence to suggest that a policy of TGC may be beneficial to neonates and children undergoing neonatal and paediatric intensive care, but none of this evidence is from large rigorous randomized controlled trials. The aim of the present study is to determine whether a policy of strictly controlling BG using insulin in children admitted to paediatric intensive care reduces mortality, morbidity and is cost-effective.

## Methods/Design

### Study Design

**Figure 1:**
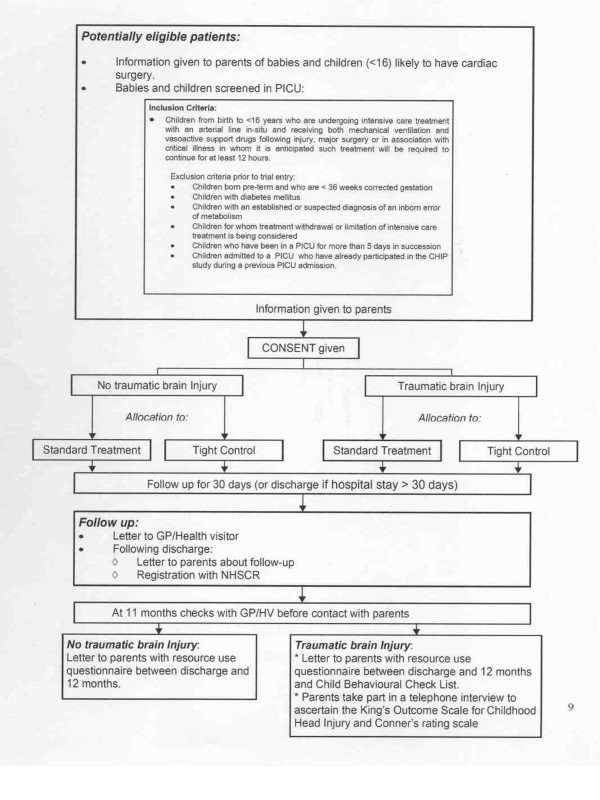
**Flow diagram of the CHiP Trial Protocol**.

The study is an individually randomised controlled open trial with two parallel groups. The protocol is summarised in Figure 1.

#### Main hypothesis

For children aged from birth to ≤ 16 years on ventilatory support and vasoactive support drugs, tight glucose control (TGC) will increase the numbers of days alive and free of mechanical ventilation at 30 days.

#### Secondary hypotheses

That TGC will lead to improvement in a range of complications associated with intensive care treatment and be cost effective.

### Setting

The following PICUs in the United Kingdom (UK) will be recruiting patients into the CHiP trial: Birmingham Children's Hospital; Bristol Royal Hospital for Children; Great Ormond Street Hospital; Leeds General Infirmary; University Hospitals of Leicester - Glenfield Hospital; Royal Brompton & Harefield NHS Trust (Royal Brompton Hospital); Royal Liverpool Children's NHS Trust; Royal Manchester Children's Hospital; St Mary's Hospital; Sheffield Children's NHS Foundation Trust; Southampton General Hospital; University Hospital of North Staffordshire.

### Ethical Approval

MREC approval obtained from the Brighton East Research Ethics Committee (re 07/Q1907/24) in 2007 and SSIs have been successfully completed for all 10 participating centres. Over 500 children have thus far been recruited.

### Type of participants

#### Inclusion criteria

Children from birth (≥ 36 weeks corrected gestation) to ≤ 16 years who are undergoing treatment on a PICU with an arterial line in-situ and who are receiving both mechanical ventilation and vasoactive drugs (Table 1) following injury, major surgery or in association with critical illness in whom it is anticipated that such treatment will be required to continue for at least 12 hours.

**Table 1:** Definition of vaso-active drugs

Vaso-active drug name	Dose
Dobutamine	> 5 mcg/kg/min
Dopamine	> 5 mcg/kg/min
Epinephrine	Any dose
Norepinephrine	Any dose
Milrinone	Any dose
Vasopressin	Any dose

#### Exclusion criteria prior to trial entry

• Children born pre-term and who are < 36 weeks corrected gestation

• Children with diabetes mellitus

• Children with an established or suspected diagnosis of an inborn error of metabolism

• Children for whom treatment withdrawal or limitation of intensive care treatment is being considered

• Children who have been in a PICU for more than 5 days

• Children admitted to a PICU who have already participated in the CHIP study during a previous P**I**CU admission.

#### Consent

Parents/guardians of babies and children in intensive care are likely to be stressed and anxious. However they will be asked to give consent in their role of legal representatives and will usually have limited time to consider trial entry as it may not be medically appropriate to delay the start of treatment. Parents of babies and children listed for cardiac surgery will be given information about the trial pre-operatively and consent provisionally obtained to be confirmed later if the child is admitted to intensive care. In addition, where possible, older children will be given information and asked to assent to their participation in the study.

### Patients not entered into the trial will receive standard care

#### Allocation

To reduce the risk of selection bias at trial entry, allocation will be administered through a 24 hour, 7 day a week central randomisation service. Minimisation with a probabilistic element will be used to ensure a balance of key prognostic factors between groups using the following criteria:

• Centre

• Age ≤ 1 year versus between 1 year and ≤ 16 years

• Admitted following cardiac surgery or not

• For cardiac surgical children, Risk adjusted classification for Congenital Heart Surgery 1 (RACHS1)[49] category 1 to 4 versus 5 to 6

• For non-cardiac surgical children, Paediatric index of mortality version 2 (PIM2) score at randomization categorised by probabilities of death of <5%, 5% - <15% and ≥ 15%

• Accidental TBI or not

### Interventions

After inclusion in the study, children will be randomised to one of two groups: Group 1 (Standard treatment) or Group 2 (Tight glycaemic control).

#### Group 1 - Standard treatment

**Figure 2:**
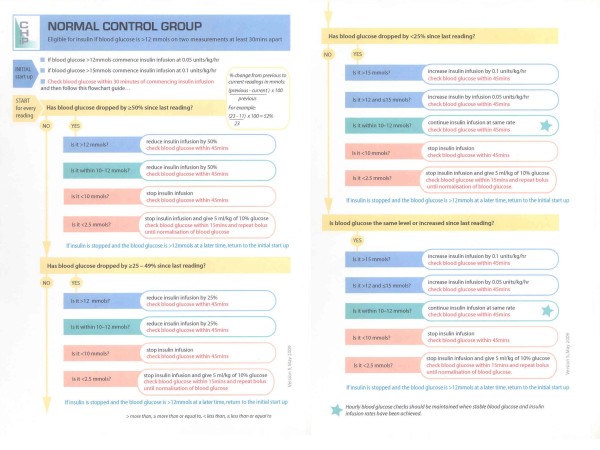
**Algorithm for the titration of insulin in the normal control group**.

Children in this group will be treated according to a standard, current, approach to BG management. Insulin will be given by intravenous infusion in this group only if BG levels exceed 12 mmol/l on two blood samples taken at least 30 minutes apart and will be discontinued once BG falls to <10 mmol/l. A protocol for glucose control in this group is in Figure 2.

#### Group 2 - Tight glycaemic control

**Figure 3:**
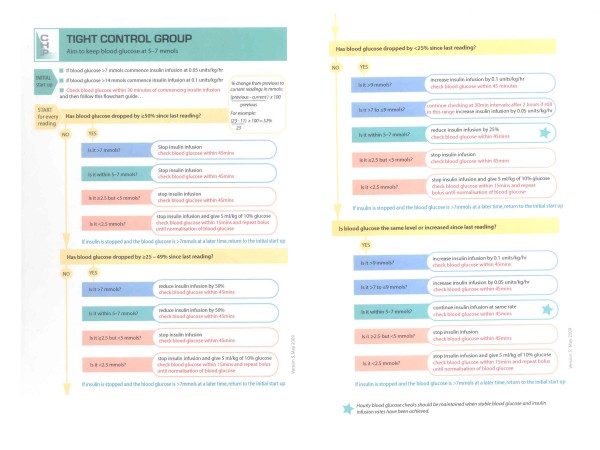
**Algorithm for the titration of insulin in the tight control group**.

Children in this group will receive insulin by intravenous infusion titrated to maintain a BG between the limits of 4 and 7.0 mmol/l. A protocol for glucose control in this group is in Figure 3.

The protocol for glucose control in group 2 has been carefully designed to achieve tight glucose control whilst minimizing the risk of hypoglycaemia, the principal side effect of insulin therapy. Standard insulin solutions will be used and changes in insulin infusion rates will be guided both by the BG and its rate of change from previous measurements. BG levels will be routinely measured as in all PICUs using commercially available 'point of care' analysers which utilise very small blood samples, producing results in approximately 1 minute. Analysers are rigorously maintained and subjected to laboratory-standard quality assurance programmes.

Training in use of the BG control protocol will be provided before the first patient is enrolled in each collaborating centre and for new staff throughout the trial. The Clinical Co-ordinating centre team will liaise closely with local clinicians to ensure that BG control algorithms are followed closely and safely.

### Outcome measures

#### Primary outcome

Following the influential ARDSNET study[50] the primary outcome for CHiP trial is the number of days alive and free from mechanical ventilation within the 30 days after trial entry. Death is obviously an important outcome. Mechanical ventilation can be seen as a measure of disease severity, defining the need for complex intensive care. The concept of ventilator free days (VFDs) brings together these two outcomes. Schoenfeld[51] define VFDs as: VFD = 0 if the child dies before 30 days; VDF = (30-x) if the child is successfully weaned from ventilator within 30 days (where × is the number of days on ventilator); or VFD = 0 if the child is ventilated for 30 days or more. The use of organ failure free days to determine patient-related morbidity surrogate end-points in paediatric trials has been supported by influential paediatric trialists in the current low mortality paediatric critical care environment[52].

#### Secondary outcomes

Death within 30 days after trial entry (or before discharge from hospital if duration is greater than 30 days)

Death within 12 months of trial entry

Number of days in PICU

Duration of mechanical ventilation

Duration of vasoactive drug usage (adrenaline, noradrenaline, dopamine, dobutamine, or Phoshopdiesterase type III [PDEIII] inhibitors or vasopressors)

Need for renal replacement therapy

Blood stream infection (positive cultures associated with two or more features of systemic inflammation or any positive blood culture for bacteria or fungi)

Use of antibiotics >10 days

Number of red cell transfusions

Number of hypoglycaemic episodes moderate (≤ 2.5 mmol/L), severe (≤ 2.0 mmol/L)

Occurrence of seizures (clinical seizures requiring anticonvulsant therapy)

Paediatric logistic organ dysfunction (PELOD) score[52-54],

Hospital length of stay

Number of children readmitted to PICU within 30 days of trial entry

#### Cost and cost-effectiveness measures

Hospital costs within 30 days of trial entry

Cost per life year (based on 30 days costs and survival)

Hospital and community health service costs within 12 months of trial entry

Cost per life year (based on 12 month costs and survival for all cases)

Cost per disability-free survivor (based on 12 month cost and outcome data for sub group with traumatic brain injury)

#### Follow-up at 12 months

If parents give their consent, all children surviving to hospital discharge will be followed up to 12 months post-randomisation to determine mortality using the NHS Central Register of the Office of National Statistics (ONS). Parents will be informed about the follow-up study at trial entry and asked to give consent. The Trial Manager at the Data Co-ordinating Centre (DCC) will write to parents following discharge home to remind them about the follow-up and ask them to keep the DCC informed about any change of address. At hospital discharge parents will be given a diary to help them record their child's service use post discharge. At around 11 months, following checks with the GP/Health Visitor to determine that this is appropriate, the Trial Manager will send a questionnaire to parents to determine the use of health care resources between discharge and 12 months. Non-responders will be followed-up by letter and telephone.

#### Follow-up of traumatic brain Injury sub-group

TBI is defined for this study as accidental trauma to the head resulting in need for intubation and mechanical ventilation. There are approximately 750 ICU admissions per year in the UK, and an estimated 150 will be recruited into CHiP.

This sub-group is more likely to have longer-term morbidity and parents of children (aged 4 or over) in this sub-group will be asked to provide additional information at 12 months, regarding overall health status, global neurological outcome, attention and behavioural status.

Outcome assessment will comprise four components:

Overall health status: measured by the Health Utilities Index (HUI)

Global neurological outcome: measured by the Kings Outcome Scale for Childhood Head Injury (KOSCHI)

Attention and behavioural assessment: measured by the Child Behavioural Check List (CBCL) and the Connor's Rating Scales revised - short version (CRS-R:S)

The HUI and KOSCHI will be completed using a structured telephone interview (around 10 minutes). The CBCL and CRS are both written questionnaires that will be posted out to the families. They take approximately 30 minutes to complete.

The Health Utilities Index is a multi-attribute health status classification system. Seven attributes (sensation, mobility, emotion, cognition, self-care, pain, fertility) are categorised according to one of 4 or 5 levels. In this population fertility will be excluded. The algorithm (from death to perfect health scale) provides a single numerical value.

KOSCHI is a 5 point categorical scale, ranging from death to normal neurological function, and is similar in structure to the Glasgow Outcome Scale, which is widely used in adult studies. In addition the KOSCHI is further subdivided into two subcategories at points 4 and 5 on the scale (moderate outcome and good outcome). Patient outcomes will be dichotomized between patients in categories 1, 2, 3, 4A and those in 4B, 5A, 5B.

Child behaviour checklist (CBCL/4-18) (problem scales) is based on parental report and assesses problematic child behaviour that is summarised in internalising behaviour (anxious/depressed, withdrawn/depressed, somatic complaints), externalising behaviour (rule-breaking, aggressive) and other (social problems, thought problems, attention problems).

In reference to 1991 normative data (Table 2) Patient outcome can be summarised according to placement within one of the three groups, or according to the T-score.

**Table 2:** Child behavior checklist (CBCL/4-18) assessment of outcome according to T-score

T-score (Whole)	Guideline	T-score (Individual scale)	Guideline
<60	Normal	<65	Normal
60-63	Borderline	65-69	Borderline
>63	Clinical	>69	Clinical

The Conners' rating scales (revised - short version CRS-R:S) assesses symptoms of attention-deficit/hyperactivity disorder and related problem behaviour in children and adolescents based on parent's report.

In reference to 1993 normative data (Table 3) Patient outcome can be summarised according to placement within one of the three groups (marked + moderate, mild + slight, average + good), or according to the T-score.

**Table 3:** The Conner's' rating scales (revised - short version CRS-R:S) assessment of outcome according to T-score

T-score	Guideline
≥ 70	Markedly atypical (significant problem)
66-69	Moderately atypical (significant problem)
61-65	Mildly atypical (possible significant problem)
56-60	Slightly atypical (borderline)
45-55	Average (no concern)
≤ 44	Good

### Adverse events and safety reporting

The Royal Brompton & Harefield NHS Trust, as sponsor of this study, has responsibility to ensure arrangements are in place to record, notify, assess, report, analyse and manage adverse events in order to comply with the UK regulations of Medicines for Human Use (Clinical Trials) Regulations 2004.

All sites involved in the study are expected to inform the Chief Investigator and Study nurse of any serious adverse events/reactions within 24 hours so that appropriate safety reporting procedures can be followed by the Sponsor.

#### Expected side effects

All adverse events judged by either the investigator or the sponsor as having a reasonable suspected causal relationship to insulin therapy qualify as adverse reactions.

Whilst any suspected, unexpected, serious adverse reaction (SUSAR) involving insulin therapy will be reported according to the timelines for SUSARs, expected side effects of insulin will be reported in the annual safety report unless serious enough to warrant expedited reporting.

The most prominent adverse effect of insulin treatment is hypoglycaemia. This is particularly important in the TCG arm of the study which is aiming to control BG within the range 4 - 7 mmol/l which is well above the 2 mmol/l threshold for clinically important hypoglycaemia [55]. The principal measure to avoid clinically important hypoglycaemia will be hourly measurement of BG when insulin is first administered. The insulin administration protocols aim to achieve glucose control with the lowest possible incidence of hypoglycaemia and the avoidance of neuroglycopaenia. Hypoglycaemic events will be reported to the Clinical Co-coordinating Centre and if necessary, the BG control protocols will be revised, whilst still aiming to achieve BG levels within the target ranges.

Insulin is reported to occasionally cause a rash which may be associated with itching.

### Data collection

To minimise the data collection load for busy units, the trial will collaborate with the Paediatric Intensive Care Audit Network (PICANet http://www.picanet.org.uk) to make best use of the established data collection infrastructure which exists in all PICUs in the UK. The PICANet dataset includes most of the items being used in the trial and these data will be transmitted from the participating centres to the Data Co-ordinating Centre electronically using strong encryption. The remaining short term data items will be collected locally by the research nurses, and those for the longer term follow-up will be collected separately by telephone and postal questionnaires. These data will be double entered onto electronic database storage systems at the Data Co-ordinating Centre.

### Economic evaluation

Cost-consequence and cost-effectiveness analyses will be undertaken as part of the proposed study. These economic evaluations will assess whether the costs of achieving tight BG control are justified by subsequent reductions in hospitalisation costs and/or by improvements in patient outcomes. The evaluations will be conducted in two phases, in the first phase all hospital costs at 30 days post randomisation will be compared across treatment groups alongside 30-day outcomes, in the second phase cost and outcomes at 12-months will be compared across the groups.

For the first phase evaluations, detailed resource use data will be collected for each patient enrolled in CHIP using the Paediatric Critical Care Minimum Dataset (PCCMDS)[56] which will be collected by each PICANet unit. Where information on resource use required in CHIP is not available from these sources datasheets similar to those developed as part of the INNOVO study will be used [57]. Information will also be collected on the resources required to achieve tight BG control, in particular all medication use and the staff time involved in monitoring the patients and managing adverse events (e.g. hypoglycaemia) will be noted.

Unit costs for hospital services will be taken from the NHS 'payment by results' database[58]. Where more detailed unit costs are required, for example those associated with staff time and the use of insulin infusion, these will be collected on site visits to centres. Hospital costs up to 30 days will be estimated by valuing each resource use item by the appropriate unit cost.

In the second phase of the study the time horizon of the economic evaluation will be extended to 12 months, and resource use data for hospital re-admissions, outpatient visits and the use of community health services will be collected for all cases. For the sub-sample of patients diagnosed as having traumatic brain injury at study entry, information on the patient's disability at one-year will be collected during telephone interviews with the patients' relatives based on previously developed interview schedules [57]. All community service use will be valued using national unit costs[59]. Total costs for each patient will be calculated by summing the costs of all hospital and community health services used.

All the economic analyses will be based on the treatment groups as randomly allocated ('intention to treat'). The initial analysis will include a cost-consequence analysis and will report mean differences (95% CI) between treatment groups in resource use (e.g. length of hospital stay) and total hospital costs per patient, alongside the primary clinical endpoint. The initial analysis will also combine costs and outcomes at 30 days post-randomisation in a cost-effectiveness analysis, which will report cost per death averted and cost per adverse event averted. The subsequent analysis will use 12-month cost and outcome data to report the cost per death averted for all patients. For the sub-sample of patients diagnosed as having brain injury at study entry, the cost-effectiveness analysis will also report the cost per death or disabled case averted.

The sensitivity analysis will test whether the results are robust to key assumptions made, for example to the choice of unit costs and the time horizon of the analysis. The cost and outcome data collected at one-year will be used to project the impact of the intervention on longer-term costs and outcomes.

### Sample size

A difference of 2 days in the number of ventilator-free days (VFD) within the first 30 days post-randomisation between the two groups has been chosen as the primary outcome measure for the trial. Information from PICANet using data from UK PICUs for 2003-4 estimates that the mean number of VFDs in cardiac patients is 26.7, with a standard deviation (SD) of 4.2. Corresponding figures for non-cardiac patients are a mean of 22.7 days, with a standard deviation (SD) of 6.8 days. As the SD is estimated with error, to be conservative we have assumed the SD is nearer 5.5 days for the cardiac and 8 days for the non-cardiac patients. There are likely to be more non-cardiac than cardiac patients eligible for the trial. We have therefore assumed an overall SD across both cardiac and non-cardiac strata of 7 days. Assuming this is the same in both trial arms, and taking a type I error of 1% (with a 2-sided test), a total sample size of 750 patients would have 90% power to detect this difference. Whereas we can assume minimal loss to follow up to 30 days, there may be some non-compliance (some patients allocated to tight control not receiving this, and some allocated to usual care being managed with tight control). The target size will therefore be inflated to 1000 to take account of possible dilution of effect.

As information from PICANet indicates that there are differences in outcome between cardiac and non-cardiac patients not merely in VFDs but also in 30 day mortality rate (3.4% vs. 20%) and mean duration of time on a ventilator (3.7 vs. 8.0 days, survivors and non-survivors combined), we also wish to be able to detect whether any effect of tight glucose control differs between the cardiac and non-cardiac strata. To have 80% power for an interaction test to be able to detect a difference of two days in the effect of intervention between the strata at the 5% level of statistical significance, we would need to increase the sample size to 1500. If the interaction test was positive this size would allow us to assess the effect of tight glucose control separately in the two strata.

#### Recruitment rate

There are approximately 1300 cardiac and 1550 non-cardiac eligible patients per year in the collaborating PICUs. If half of those eligible are recruited into the trial, it should be feasible to recruit the overall total sample size of 1500 within the 24 months recruitment period.

### Type of analysis

Analysis will be by intention to treat. The following sub-group analyses will be conducted; age (Age ≤ 1 year versus between 1 year and ≤ 16 years), severity of illness, traumatic brain injury or not, cardiac surgical versus non-cardiac cases, RACHS1 (cardiac cases) (Groups 1-4 versus 5 and 6), PIM2 group (non-cardiac cases) (categorised by probabilities of death of <5%, 5% - <15% and ≥ 15%), run in cases v. non-run in cases.

#### Frequency of analysis

An independent Data Monitoring and Ethics Committee (DMEC) will review, in strict confidence, data from the trial approximately half way through the recruitment period. The Chair of the DMEC may also request additional meeting/analyses. In the light of these data and other evidence from relevant studies, the DMEC will inform the Steering Committee if in their view:

i. There is proof that the data indicate that any part of the protocol under investigation is either clearly indicated or clearly contra-indicated either for all patients or a particular subgroup of patients. using the Peto and Haybittle rule [60,61]

ii. It is evident that no clear outcome will be obtained with the current trial design.

iii. That they have a major ethical or safety concern

### Ancillary studies

In addition to the main study, some collaborators may wish to conduct other more detailed or complementary studies. The grant holders welcome this provided that proposals are discussed in advance with the Trial Steering Committee and appropriate additional Research Ethics approval is sought.

### Organisation

The Trial Steering Committee (TSC) responsibilities are to approve the main study protocol and any amendments, monitor and supervise the trial towards its interim and overall objectives, review relevant information from other sources, consider the recommendations of the DMEC, and resolve problems brought by the trial co-coordinating centres. Day to day management of the trial will be overseen by a Trial Management Group (TMG) comprising the grant holders and project staff from the Clinical Co-coordinating Centre at the Royal Brompton Hospital NHS Trust and the Data Co-coordinating Centre (DCC) at the LSHTM.

### Publication policy

To safeguard the integrity of the trial, data from this study will not be presented in public or submitted for publication without requesting comments and receiving agreement from the Trial Steering Committee. The primary results of the trial will be published by the group as a whole with local investigators acknowledged. The success of the trial depends on the collaboration of many people. The results will be presented first to the trial local investigators. A summary of the results of the trial will be sent to parents of participating children on request and also made available on the trial website.

### Confidentiality

Patients will be identified by their trial number to ensure confidentiality. However, as the patients in the trial will be followed up to 12 months following randomisation, it is essential that the team at the Data Co-coordinating Centre has the names and addresses of the trial participants recorded on the data collection forms in addition to the allocated trial number. Stringent precautions will be taken to ensure confidentiality of names and addresses at the Data Co-coordinating Centre.

The Chief Investigator and local investigators will ensure conservation of records in areas to which access is restricted.

### Audit

To ensure that the trial is conducted according to ICH GCP guidelines, site audits will be carried out on a random basis. The local investigator will be required to demonstrate knowledge of the trial protocol and procedures and Good Clinical Practice. The accessibility of the site file to trial staff and its contents will be checked to ensure all trial records are being properly maintained. Adherence to local requirements for consent will be examined.

If the site has full compliance the Site Visit Form will be signed by the Trial Manager. In the event of non-compliance the Data Coordinating Centre will address the specific issues to ensure that relevant training and instruction is given.

### Termination of the study

At the termination of planned recruitment the Data Co-coordinating Centre will contact all sites by telephone, email or fax in order to terminate all patient recruitment as quickly as possible. If the study is terminated prematurely by the Steering Committee all sites will be informed immediately. When all recruited patients have been followed until 30 days post randomisation (or hospital discharge if stay longer than 30 days) a declaration of the end of trial form will be sent to EurdraCT and the MREC. The following documents: original consent forms, original data forms, trial related documents and correspondence will be archived in each Site File and kept for at least five years. At the end of the analysis and reporting phase, the Trial Master Files at the Clinical and Data Co-coordinating Centres will be archived for 15 years.

### Indemnity

If there is negligent harm during the clinical trial when the NHS body owes a duty of care to the person harmed, NHS Indemnity covers NHS staff, medical academic staff with honorary contracts, and those conducting the trial. NHS Indemnity does not offer no-fault compensation.

## Discussion

Data from level 2 trials have driven the adult intensive care clinicians to adopt treatment regimes that favour tight glycaemic control. Equipoise presently exists in the paediatric intensive care community and this allows us a very important opportunity to conduct an adequately powered randomised controlled trial in this setting.

## Abbreviations

AE: Adverse Event; AR: Adverse Reaction; BG: Blood glucose; CBCL: Child Behavioural Check List; CHiP: Control of Hyperglycaemia in Paediatric Intensive Care; DCC: Data Co-coordinating Centre; CI: Confidence interval; CRS-R:S: Connor's Rating Scales revised - short version; DMEC: Data Monitoring and Ethics Committee; GCP: Good Clinical Practice; HTA: Health Technology Assessment; HUI: Health Utilities Index; ICNARC: Intensive Care Audit and Research Network; JACHO: Joint Commission on Accreditation of Healthcare Organization; KOSCHI: Kings Outcome Scale for Childhood Head Injury; LSHTM: London School of Hygiene and Tropical Medicine; NEC: Necrotising enterocolitis; ONS: Office of National Statistics; PCCMDS: Paediatric Critical Care Minimum Dataset; PELOD: Paediatric logistic organ dysfunction; PDEIII: Phoshodiesterase type III; PI: Principal investigator; PICANet: Paediatric Intensive Care Audit Network; PICU: Paediatric intensive care unit; PIM2: Paediatric index of mortality version 2; RACHS1: Risk adjusted classification for Congenital Heart Surgery 1; RR: Relative risk; SD: Standard deviation; SPC: Summary of Product Characteristics; SSAR: Suspected Serious Adverse Reaction; SUSAR: Suspected, unexpected, serious adverse reaction; TBI: Traumatic brain injury; TGC: Tight glucose control; TMG: Trial Management Group; TSC: Trial Steering Committee; VFD: Ventilator free days

## Competing interests

The authors declare that they have no competing interests.

## Authors' contributions

All authors have read and approved the final manuscript.

DM came up with original concept; RT on behalf of the Paediatric Intensive Care Society Study Group endorsed the proposal; DM, RT, RG, RP and DE wrote the grant application and original protocol, JP has written this submission; All other authors played important parts in final protocol development.

## Authors' information

### Trial steering group

Professor Michael Preece (Chair) Consultant Paediatrician, Great Ormond Street Children's Hospital; Mrs. Pamela Barnes Lay member; Ms Sian Edwards Paediatric Pharmacist, Royal Brompton Hospital; Professor David Field Neonatologist, Leicester Royal Infirmary and the University of Leicester; Dr. James Hooper Consultant Clinical Biochemist, Royal Brompton Hospital; Mrs. Tara Quick Lay member, Parent; Dr Claire Snowdon Centre for Family Research, University of Cambridge; Ms Lyvonne Tume Research Nurse, Royal Liverpool Children's Hospital; Dr. Dirk Vlasselaers Consultant Paediatric Intensivist, Leuven, Belgium; Professor Paula Williamson Professor of Medical Statistics, University of Liverpool.

(In attendance: Mr. Michael Loveridge Royal Brompton Hospital (Trial sponsor))

### Data monitoring committee

Professor David Dunger (CHAIR), Department of Paediatrics, University of Cambridge; Dr David Harrison, Statistician, Intensive Care Audit and Research Network (ICNARC); Professor David Hatch, Emeritus Professor of Paediatric Anaesthesia and Intensive Care, Great Ormond Street Hospital; Mr. Giles Peek, Consultant Cardiac Surgeon, Glenfield Hospital, Leicester (until 2009);

Dr Jon Smith, Consultant Paediatric Cardiothoracic Anaesthetist, Freeman Hospital, Newcastle (from 2009).

## Pre-publication history

The pre-publication history for this paper can be accessed here:

http://www.biomedcentral.com/1471-2431/10/5/prepub
